# Regulatory T cells in ischemic stroke

**DOI:** 10.1111/cns.13611

**Published:** 2021-01-20

**Authors:** Huan Wang, Zhao Wang, Qianqian Wu, Yujia Yuan, Wen Cao, Xiangjian Zhang

**Affiliations:** ^1^ Department of Neurology Second Hospital of Hebei Medical University Shijiazhuang Hebei PR China; ^2^ Hebei Collaborative Innovation Center for Cardio‐cerebrovascular Disease Shijiazhuang Hebei PR China; ^3^ Hebei Vascular Homeostasis Key Laboratory Shijiazhuang Hebei PR China

**Keywords:** immunomodulation, ischemic stroke, neuroinflammation, neuroplasticity, regulatory T cells

## Abstract

The pathophysiological mechanisms of neuroinflammation, angiogenesis, and neuroplasticity are currently the hotspots of researches in ischemic stroke. Regulatory T cells (Tregs), a subset of T cells that control inflammatory and immune responses in the body, are closely related to the pathogenesis of ischemic stroke. They participate in the inflammatory response and neuroplasticity process of ischemic stroke by various mechanisms, such as secretion of anti‐inflammatory factors, inhibition of pro‐inflammatory factors, induction of cell lysis, production of the factors that promote neural regeneration, and modulation of microglial and macrophage polarization. However, it remains unclear whether Tregs play a beneficial or deleterious role in ischemic stroke and the effect of Tregs in different stages of ischemic stroke. Here, we discuss the dynamic changes of Tregs at various stages of experimental and clinical stroke, the potential mechanisms under Tregs in regulating stroke and the preclinical studies of Tregs‐related treatments, in order to provide a reference for clinical treatment.

## Graphical abstract

This review is more comprehensive and specific summarizes the dynamic changes and potential mechanisms of Tregs in the peripheral and brain after ischemic stroke, and Tregs‐related treatments in stroke. To help researchers gain an overall understanding of Tregs in stroke.

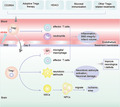

## 1 INTRODUCTION

Ischemic stroke, which usually leads to death and long‐term disability, is one of the major threats to human health. Recombinant plasminogen activator (rt‐PA) remains the only FDA approved pharmacological treatment for ischemic stroke, but its use is limited by the narrow time window and the risk of bleeding. Therefore, finding new and effective treatments is essential for improving neurological function. The immune system plays an important role in the pathophysiological process of ischemic stroke. After stroke, inflammatory processes are activated, including neuronal necrosis, blood‐brain barrier (BBB) disruption, microglia activation, leukocyte infiltration, and inflammatory factors release.^1^ Regulatory T cells (Tregs) are a specialized subpopulation of T lymphocytes, which participate in the post‐stroke immune response through a variety of mechanisms. This review summarizes the mechanisms of Tregs in stroke and Tregs‐related treatment of stroke.

## 2 OVERVIEW AND BIOLOGICAL CHARACTERISTICS OF TREGS

Tregs, specifically expressing the intracellular transcription factor Forkheadbox P3 (FoxP3), play an immunosuppressive role in various diseases.^2^, ^3^ Tregs can be generally divided into two subsets. The first one is directly derived from the thymus, called thymus‐derived Treg (tTreg) or naturally occurring Treg (nTreg), whose differentiation is the result of the high‐affinity interaction with autopeptide/MHC II complex during T‐cell development within the thymus.^4^ The other one is peripheral‐derived Treg (pTreg),^5^ which is differentiation from naive CD4^+^ T cell in the periphery upon antigen stimulation with an appropriate combination of cytokines, including interleukin (IL)‐2 and transforming growth factor‐β (TGF‐β).^6^ By regulating the function of effector T cells and secreting anti‐inflammatory factors such as IL‐10 and TGF‐β,^7^, ^8^ Tregs are crucially involved in the modulation of basal immunity, maintenance of immune homeostasis, and regulation of immune response to diseases.^9^

Tregs are present in both lymphoid and non‐lymphoid tissues in the physiological state. Defense is the primary role of Tregs in lymphoid tissue, while homeostasis is mainly maintained by Tregs localized in non‐lymphoid parenchymal tissues,^10^ called tissue Tregs. Tissue Tregs can be found in skin, muscle tissue, lung, colon, visceral fat tissue, and brain.^11^ They not only have immunosuppressive function, but also accumulate after tissue injury, playing an important role in regulating the local inflammatory response, promoting tissue repair and improving aging and obesity.^11^

Tregs exhibit common properties among tissues, although they also have characteristics specific to each tissue.^10^ Tregs phenotypically diverse,^12^ determined by different cell surface markers.^13^ Foxp3 is an X chromosome‐related factor essential for the development and management of the inhibitory function of Tregs, and its expression level determines the immunosuppressive properties of Foxp3^+^ Tregs.^14^ Almost all inhibitory Tregs express Foxp3.^13^ CD25, a component of high‐affinity IL‐2 receptor, is expressed on the surface of almost all Tregs and is required for Tregs’ survival.^15^ IL‐2 can stabilize Foxp3 expression and regulate the production of Tregs surface molecules such as cytotoxic T lymphocyte‐associated antigen‐4 (CTLA‐4) and tumor necrosis factor receptor (TNFR) by inducing Foxp3 mRNA production to maintain cell stability. Tregs in brain resemble other tissue Tregs in many ways, but brain Tregs express genes unique to central nervous system, such as serotonin receptor type 7 (Htr7). The expansion of brain Tregs is dependent on IL‐2, IL‐33, serotonin, and T‐cell receptor (TCR) recognition. The penetration of Tregs into the brain is driven by chemokine (C‐C motif) ligand 1 (CCL1) and chemokine (C‐C motif) ligand 20 (CCL20). Brain Tregs also express higher levels of CTLA‐4, CD130, CD39, amphiregulin (AREG), and glucocorticoid‐induced tumor necrosis factor receptor family‐related gene (GITR), ST2 (IL‐33 receptor subunit), which are also expressed in other tissue Tregs.^16^

## 3 DYNAMIC CHANGES OF TREGS IN VARIOUS STAGES OF EXPERIMENTAL ISCHEMIC STROKE

### 3.1 Changes in the number of Tregs in the brain after ischemic stroke

It was found that the number of CD25^+^ Foxp3^+^ Tregs in the ischemic area accounted for less than 5% of CD4^+^ T cells during the first week after transient middle cerebral artery occlusion (tMCAO)^17^ and started to increase to about 20% of CD4^+^ T cells on the 7^th^ to 10^th^ day after stroke.^18^ On day 14 after stroke, 40% of CD4^+^ T cells were Foxp3^+^ Tregs, which were recruited in and around the infarct area, and their number persistently increased and remained at a high level for 2 months.^16^ Tregs may proliferate on the ischemic hemisphere side 7 days after tMCAO, indicating a possible kinetic delay in the adaptive immune response.^16^, ^18^, ^19^ The increase of brain Tregs was significantly higher in aged male mice than in females at 15 days after tMCAO, indicating that the immune response of brain T cells may be time‐specific and gender‐specific.^20^ In contrast to these findings, Kleinschnitz et al. found a marked increase of Foxp3^+^ Tregs in the brain within 24 hours after tMCAO, but predominantly in the cerebral vasculature.^21^ Moreover, on day 3 after permanent middle cerebral artery occlusion (pMCAO), Tregs on the ischemic hemisphere accounted for about 20% of CD4^+^ T cells, which may cause more severe neuroinflammatory reaction.^22^

### 3.2 Changes in the number of peripheral Tregs after ischemic stroke

Normally, Tregs make up only 5% to 10% of circulating T cells. After experimental stroke, the total number of peripheral blood T cells was significantly reduced, while the proportion of Tregs was significantly increased.^23^ The proportion of Tregs in the peripheral blood was dramatically downregulated on day 1 after tMCAO, returning to normal amount on day 3 and to about 10% on day 7, which indicates the redistribution of Tregs post‐stroke. It is speculated that Tregs migrate from the periphery to the brain in the early stage of stroke and exert early effects on cerebral ischemia through the peripheral immune system.^18^, ^21^

## 4 THE MECHANISMS OF TREGS IN EXPERIMENTAL STROKE

The role of Tregs in ischemic stroke is highly controversial. Most studies have proved the protective effect of Tregs in ischemic stroke.^22^, ^23^, ^24^ However, the correlation between Tregs and stroke is somewhat questioned,^18^, ^25^, ^26^ and it has been reported that Tregs aggravate ischemic brain injury.^21^

### 4.1 The protective mechanisms of Tregs in ischemic stroke

#### 4.1.1 Possible neuroprotective mechanisms of peripheral Tregs in the early stage of stroke

**FIGURE 1:**
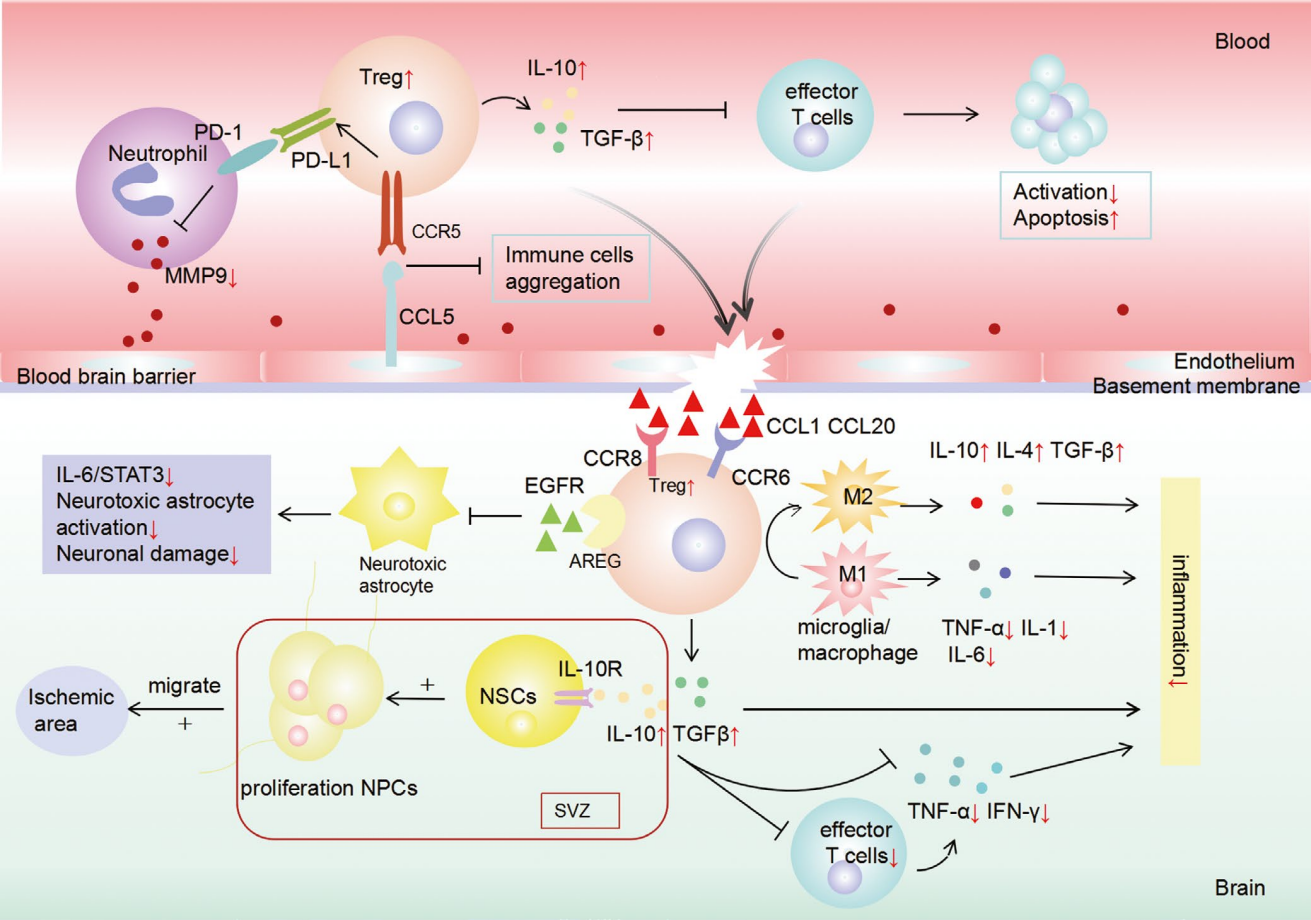
The protective mechanisms of regulatory T cells (Tregs) in ischemic stroke. Tregs play a neuroprotective role in the early stage of ischemic stroke by acting on white blood cells in peripheral blood: (1)Tregs inhibit the production of neutrophil‐derived metalloproteinase‐9 (MMP‐9) by expressing programmed death ligand‐1 (PD‐L1), thus protecting the integrity of the blood‐brain barrier (BBB). (2)Tregs can directly activate C‐C Chemokine Receptor Type 5 (CCR5) and combines with chemokine (C‐C motif) ligand 5 (CCL5) on endothelial cells to prevent other immune cells from staying in the ischemic area, thus protecting the BBB. (3)Tregs inhibit the activation of effector T cells by producing anti‐inflammatory factor such as interleukin (IL)‐10 and transforming growth factor‐β(TGF‐β). About 5–7 days after stroke, Tregs was driven to infiltrate into the brain by the chemokine (C‐C motif) ligand 1 (CCL1) and chemokine (C‐C motif) ligand 20 (CCL20) and play a brain‐protective role in the following ways: (1)Brain Tregs prevent microglia/macrophage polarization toward the M1 type and modulate microglia/macrophage polarization toward the M2 phenotype, thus reducing inflammation reaction. (2)Brain Tregs can also inhibit the activation of neurotoxic astrocyte through the amphiregulin (AREG)/epidermal growth factor receptor (EGFR) pathway to promote neurological recovery. (3)Brain Tregs alleviate neural injury and promote neurogenesis through IL‐10.

Peripheral immune activation post‐stroke is a double‐edged sword for the BBB. Activated immune cells migrate to the injured area, and cytokines, chemokines and proteases secreted by effector T cells and neutrophils propagate the inflammatory cascade and destroy the integrity of the BBB. Tregs can inhibit the activity and function of effector T cells and neutrophils.^27^ Tregs in the spleen and blood are relatively expanded in the early stage of stroke.^28^, ^29^ Within 5 to 7 days after stroke,^16^, ^30^ Tregs mainly accumulate in the blood vessels of the infarct area and the surrounding area, but cannot penetrate the BBB and infiltrate the brain tissue, indicating that Tregs may exert an immunosuppressive effect through the periphery in the early stage of stroke (Figure 1).^18^, ^31^ This may explain why only a small amount of Tregs are detected in the brain in the early stage of stroke.

##### Tregs regulate the post‐stroke immune response by neutrophil in peripheral immune system

Neutrophil‐derived matrix metalloproteinase (MMP)‐9, which is elevated in the plasma within 24 hours after MCAO, participates in the destruction of BBB by degrading extracellular matrix and plays an important role in brain edema, leukocyte infiltration, and hemorrhage.^27^, ^32^, ^33^, ^34^ In addition, tissue plasminogen activator (t‐PA) promotes neutrophil degranulation and MMP‐9 release.^35^, ^36^ Mediated through programmed death‐1 (PD‐1)/programmed death ligand‐1 (PD‐L1) interactions,^37^ Tregs specifically and potently inhibit the production of neutrophil‐derived MMP‐9 after stroke.^25^, ^34^

##### Tregs protect the BBB by interacting with C‐C Chemokine Receptor Type 5 (CCR5)

CCR5 is a chemokine receptor highly expressed on T cells and is readily expressed on Tregs in certain pathological conditions.^38^ It was reported that infusion of wild‐type (WT) Tregs but not CCR5^−/−^ Tregs attenuated brain injury after 2 hours of reperfusion within 60 minutes of tMCAO. Thus, CCR5 signaling pathway may play an important role in promoting the immunosuppressive function of adoptively transferred Tregs. Chemokine (C‐C motif) ligand 5 (CCL5), the ligand of CCR5, is highly expressed on endothelial cells in the ischemic penumbra 1 day after MCAO, and meanwhile the percentage of CCR5^+^ Tregs also significantly increased in the peripheral blood. CD4^+^ CD25^+^ Foxp3^+^ Tregs can directly activate CCR5 and combines with CCL5 on endothelial cells to prevent other immune cells from staying in the ischemic area, thus protecting the BBB. Furthermore, Treg‐induced CCR5 enhanced expression of PD‐L1, thereby inhibiting neutrophil‐derived MMP‐9.^39^ However, some studies demonstrated that inhibition of CCR5 signaling can enhance learning and memory capacity, reinforces hippocampal and cortical plasticity,^40^ and promotes the recovery of motor function in early stage of stroke.^41^

##### Tregs inhibit the activation of effector T cells

T‐cell‐mediated brain injury begins to manifest 24 hours after tMCAO. Effector T cells can produce pro‐inflammatory factors to increase oxidative stress and destroy the BBB after stroke. One of the potential peripheral immunosuppressive properties of Tregs is suppressing the activation of effector T cells.^42^ Lisze et al. proved that splenic Tregs can significantly inhibit the activation of effector T cells and the production of their effector IFN‐γ after stroke in vitro.^23^ Intestinal Tregs induced by dendritic cells (DCs) suppress differentiation of effector IL‐17^+^ γδ T cells through IL‐10, inhibit effector T cells migration from the gut to the leptomeninges after stroke, and reduce chemokine expression and leukocyte infiltration.^43^ Besides, Tregs can also suppress effector T cell in the brain. A delayed and long‐term invasion of T cells into the brain has been found after stroke. Secretion of IFN‐γ, IL‐17, tumor necrosis factor‐α (TNF‐α) and perforin by brain invading T cells was significantly increased in ischemic hemisphere 3 days after stroke.^44^ Lisze's study proved that Tregs exerted a restrictive effect on the activation and IFN‐γ production of brain invading T cells 5 days post‐MCAO.^22^ Therefore, Tregs are able to suppress both peripheral and intracerebral effector T cells after stroke.

#### 4.1.2 Possible roles and mechanisms of brain Tregs in non‐early stage of stroke

About 5–7 days after stroke, Tregs was driven to infiltrate into the brain by the chemokines CCL1 and CCL20 produced by astrocytes and oligodendrocytes. C‐C Chemokine Receptor Type 8 (CCR8) and C‐C Chemokine Receptor Type 6 (CCR6) derived by Tregs bind to CCLI (CCR8 ligand) and CCL20 (CCR6 ligand), promoting the infiltration of Tregs into the brain (Figure 1).^16^, ^30^

##### Brain Tregs alleviate neural injury and promote neurogenesis through IL‐10

IL‐10 is an anti‐inflammatory factor.^45^ Studies have demonstrated that increasing the production of lymphocyte‐derived IL‐10 or intracerebral injection of IL‐10 can downregulate the neuroinflammation and reduce the cerebral infarct volume.^23^, ^46^, ^47^ IL‐10 derived from Tregs is a key mediator of cerebral protection effect post‐stroke and suppresses pro‐inflammatory cytokine production such as TNF‐α and INF‐γ.^22^, ^48^ The anti‐inflammatory effect of IL‐10 might be mediated by direct action on brain cells. On approximately 5 days after stroke, the expression of IL‐10 in brain significantly increases and is correlated with the invasion of peripheral Tregs to the brain.^23^ IL‐10 may be a critical mediator for the protection elicited by endogenous Tregs against late stages of ischemic injury, but administration of exogenous Tregs did not change IL‐10 level in the brain.^25^

In addition, IL‐10 has growth factor‐like function and has been shown to modulate neurogenesis via interacting with IL‐10 receptor (IL‐10R) expressed on proliferating neural stem cells (NSCs) and activating IL‐10‐regulated downstream signaling pathway extracellular signal‐regulated kinase (ERK) and STAT3.^49^, ^50^ Wang et al. confirmed that intracerebral injection of exogenously activated Tregs after stroke promoted NSC proliferation in the ischemic subventricular zone and increased the number of Brdu (+) cells through IL‐10.^51^ Neural progenitor cells (NPCs) migrate to the injury area and differentiate into mature neurons to replace the injury neurons.^52^ Depleting Tregs or blocking IL‐10 can inhibit NSC proliferation and NPC migration after stroke, thereby inhibiting neurogenesis. Both endogenous and exogenous Tregs can promote neurogenesis, but Tregs‐induced neurogenesis requires long‐term observation.^51^, ^53^

##### Brain Tregs suppress neuroinflammatory by interacting with microglia/macrophages

Microglia/macrophages are known to play a dual role in ischemic stroke. After cerebral ischemia, M1 phenotype microglia/macrophages rapidly activate and release a large amount of reactive oxygen species (ROS), matrix metalloproteinases (MMPs), anti‐inflammatory factors, and chemokines. Brain Tregs prevent microglia/macrophage polarization toward the M1 type and modulate microglia/macrophage polarization toward the M2 phenotype through the IL‐10/GSK3β/PTEN axis, in order to restrain the inflammatory response of macrophages and microglia and transform the balance of the microglia/macrophages reaction from cytotoxic to neuroprotective.^24^, ^54^, ^55^, ^56^ Meanwhile, M2 microglia can also promote the differentiation of Tregs to alleviate neuroinflammation.^57^ However, Shu et al. reported that microglia induced sirtuin2 expression after stroke and inhibited the anti‐inflammatory function of infiltrating Tregs.^58^

##### Brain Tregs promote neurological recovery via AREG/EGFR pathway

As a ligand of epidermal growth factor receptor (EGFR), AREG is an important key factor in tissue regeneration. EGFR activation has been shown to have a protective effect on neuronal repair in early ischemic brain injury.^59^ AREG is highly expressed by brain Tregs 14 days after stroke. Tregs can inhibit IL‐6‐STAT3 signaling pathway through the AREG/EGFR and suppress neurotoxic astrocyte proliferation,^16^, ^60^, ^61^ thereby enhancing neurological recovery. These findings indicate that Tregs are important for improving neurological symptoms in the chronic phase of stroke.

### 4.2 The deleterious mechanism of Tregs in ischemic stroke

Studies have shown Tregs may have adverse effects on the ischemic brain during the first 3 days of stroke.^22^, ^25^, ^51^ Kleinschnitz's research has exhibited that depletion of Tregs can significantly reduce infarct volume and improve cerebral reperfusion after 24 hours of stroke. However, this may not be related to the immune function of Tregs, but rather to the secondary microthrombus formation caused by Tregs.^21^, ^62^, ^63^ Tregs have a higher adhesive propensity and increase the interaction of platelets with ischemic brain endothelial cells through the LFA‐1/ICAM‐1 pathway, resulting in microvascular dysfunction, increased thrombosis and impaired reperfusion after cerebral ischemia. This may be an independent mechanism of Tregs, which can be confirmed in vitro.^21^ However, extensive experiments are still needed to prove it.

### 4.3 Tregs may also have no significant effect in ischemic stroke

The association of Tregs and ischemic stroke has been challenged by some studies, which revealed that Tregs had no significant effects on cerebral infarction volume and neurological deficit.^18^, ^22^, ^23^, ^25^, ^26^

The discrepancies in these results may be due to heterogeneity among mice, cerebral infarction volume, secondary thrombotic formation, animal model used, Treg inhibition method and inhibition efficiency, as well as the severity of cerebral ischemic injury.

## 5 TREGS IN PATIENT WITH ISCHEMIC STROKE

Tregs have not been adequately studied in stroke patients. Santamaría et al. found that circulating Tregs increased within 3 days after stroke. Patients with lower levels of circulating Tregs within 48 hours after stroke had a higher risk of neurological deterioration and infection. Circulating Tregs levels at 48 and 72 hours after stroke were negative correlated with infarct volume and independently associated with neurofunctional outcomes at 3 months.^64^ Yan's study showed that the percentage of circulating Tregs in CD4^+^ T cells increased at 1, 7, and 21 days after stroke, without significant correlation with stroke severity. The circulating Treg levels in male patients increased significantly within 3 weeks after stroke, but did not significantly change in female patients, suggesting that Tregs may be impaired in female stroke patients.^65^ It is possible that increased demethylation of Foxp3 on the X chromosome of women has an effect on Treg function.^66^ However, it was demonstrated that the level of circulating Tregs was very low within 48 hours after stroke and began to increase at day 7, which could be maintained for 3 months, suggesting that the reduction of Tregs in the early stage of stroke was not related to the development of infection or stroke outcome.^67^, ^68^ Several clinical studies have shown that the Th17/Treg ratio in peripheral blood is unstable in stroke patients.^69^, ^70^, ^71^ In addition, some studies have suggested that the proportion of circulating Tregs is positively correlated with the age of stroke patients, but not with the infarct volume.^68^, ^72^ The differential results can be caused by the following factors. Firstly, the limitations of the sample size should be assessed. The sample size of the Santamaría study was much larger than that of Urra, Yan, and Ruhnau. Secondly, the classification of cerebral infarction may be different. In the Santamaría's study, the majority were cardioembolic strokes, while TOAST classification was not performed in the other studies. Thirdly, Urra's study included some patients with hemorrhagic stroke, which may affect the results. Fourthly, the infarct volume differed. In Santamaría's study, mice had larger cerebral infarctions. Finally, Treg surface markers detected in each study were different. However, it is unclear whether the level of Tregs is related to post‐stroke infection.

## 6 TREGS‐RELATED TREATMENT IN ISCHEMIC STROKE

### 6.1 CD28 superagonist (CD28SA) affects stroke outcome by inducing Tregs amplification

CD28SA is one of the most widely used approaches to expand Tregs through endogenous mechanisms, as well as enhance their inhibitory function.^3^ CD28SA‐induced Treg expansion reduces the infarct volume after tMCAO by alleviating inflammation, and this cerebral protection mediated by IL‐10 persists till the late stage of stroke.^73^, ^74^ However, it was reported that CD28SA‐induced Treg expansion can promote vascular lesions in the acute phase of ischemic stroke and cause inflammatory thrombosis and secondary infarction, suggesting that short‐term suppression of Tregs in early stage of stroke may also be an effective treatment for ischemic stroke.^62^

### 6.2 Adoptive Tregs therapy

Adoptive Tregs therapy is the most intuitive method to expand Tregs by transfusion of purified Tregs from wild‐type animals. Adoptive transfer of Tregs can protect the tight junction and the ultrastructure of basement membrane in the early stage of stroke, thereby inhibiting the infiltration of surrounding immune cells and reducing the immunoglobulin leakage and BBB damage. Maximum brain protection can be achieved by adoptively transferring Tregs in tMCAO mice 2 hours after stroke.^25^, ^73^

Moreover, adoptive transfer of Tregs may alleviate tPA‐induced intracerebral hemorrhage (ICH) in stroke. Chemokine (C‐C motif) ligand 2 (CCL2) is a key molecule that leads to the destruction of BBB and the hemorrhagic transformation after cerebral infarction. Adoptive transfer of Tregs protects the BBB by inhibiting the production of MMP‐9 and CCL2 in tPA thrombolytic mice, significantly ameliorates inflammatory and perihematomal edema, and decreases cell death. Tregs can also improve both short‐ and long‐term outcomes by extending the treatment time window of tPA and improving the efficacy and safety of thrombolytic treatment for ischemic stroke.^75^ Therefore, the combination of tPA and Tregs for the treatment of ischemic stroke may be the direction of the future research.

However, some researchers suggest that in the early stage of ischemic stroke, adoptive transfer of Tregs does not reduce the infarct volume and improve stroke outcome, or even aggravate brain injury, and the key regulatory point of Tregs for brain protection may be in the late stage of ischemic stroke.^20^, ^21^, ^51^, ^76^

### 6.3 Histone deacetylase inhibitors (HDACi) expands Tregs

HDACi is a strong inducer of Foxp3 expression. It can improve Foxp3‐DNA binding, reduce Foxp3 turnover by ubiquitination, and convert naive T cells to a regulatory phenotype,^77^ thus increasing the number of Tregs and improving the degree of immunosuppression. The neuroprotective effect of HDACi depends on the presence of Foxp3^+^ Tregs.^23^ Additionally, HDACi can upregulate neurotrophic factors and increase the phosphorylation of STAT3, which is a key transcription factor for the activation of IL‐10 and inhibition of pro‐inflammatory cascades.^78^ These properties indicate that HDACi may be a new drug for the treatment of ischemic stroke.

### 6.4 Mucosal immunization

Mucosal immunization is the administration of cerebrovascular antigens to the mucosa, leading to the amplification of antigen‐specific Tregs that enter the central circulation and secrete immunoregulatory cytokines.^79^, ^80^ E‐selectin can be used as an immune tolerance antigen to induce immunomodulation in the ischemic damaged area and mucosal tolerance of Tregs to this antigen. Mucosal tolerance to E‐selectin can suppress activated microglia through production of TGF‐β and IL‐10 by the regulatory T cells.^81^

### 6.5 Other Tregs‐related treatments for ischemic stroke

IL‐33 is necessary for the proliferation of brain Tregs. IL‐33 receptor ST2 expressed by Tregs can modulate the expression of Foxp3 and promote the proliferation and differentiation of brain Tregs in ischemic stroke mice,^82^ thereby facilitating the proliferation of NSCs.^83^ IL‐33 combined with antibiotics can lower the risk of infection and mortality.^84^ IL‐2/IL‐2 antibody complex(IL‐2/IL‐2Ab) may induce selective expansion of Tregs and enhance their inhibitory function by blocking the binding site of IL‐2 that is needed for the expansion of other T cells after stroke, thus inhibiting neuroinflammation.^85^ Meanwhile, IL‐2Ab (JES6‐1) reduces demyelination and protects the integrity of brain white matter after ischemia stroke via suppressing CD8^+^ T cells.^86^ Knockout of acetyl‐CoA carboxylase‐1 (ACC1) gene or ACC1 inhibitor may promote the differentiation of T cells into Tregs, reduce the infarct volume and inhibit neuroinflammation without injuring the vascular structure after ischemic stroke.^87^ Some metabolites of intestinal microbiota can promote peripheral Th17/Treg balance inclining into Tregs and act on the central nervous system through the brain‐gut axis to improve the outcome of ischemic stroke. Resveratrol can achieve this effect by regulating the intestinal flora.^43^, ^88^

In addition, CXCL14 can promote the differentiation of Tregs after stroke, and passive CXCL14 supplementation may improve the stroke‐induced neuroinflammation.^89^ Rapamycin promotes Tregs to express higher levels of Foxp3 and CD25, allowing them to more strongly inhibit neuroinflammatory caused by macrophages and microglia.^24^ Serotonin or selective serotonin reuptake inhibitor (SSRI) can increase the number of brain Tregs by acting on serotonin receptors, enhance their suppression function and improve neurological symptoms.^19^, ^90^, ^91^ Hyperforin may induce Treg infiltration into the ischemic hemisphere as well as promote neuroangiogenesis and neurological function recovery, depending on IL‐6 secreted by astrocytes.^92^ Atorvastatin can enhance the inhibitory function of Tregs, induce Tregs migration to inflammatory tissue,^93^ as well as downregulate the activation of microglia and astrocytes.^94^ Persistent use of potent Poly (ADP‐ribose) polymerase‐1 (PARP‐1) inhibitors after ischemic stroke can effectively increase the proportion and suppression of Tregs by inhibiting the destruction of Foxp3 stability by PARP‐1, thereby promoting the expression of anti‐inflammatory factors.^76^, ^95^ Tregs derived from bone marrow‐derived stem cells (BMSc) also provide neuroprotective effects for ischemic stroke.^96^ Recent studies have shown that blocking Neuropilin‐1 (Nrp‐1), which is expressed on Tregs, can inhibit Nrp1‐mediated accumulation of Tregs within tumor, increase peripheral and brain Tregs, and reduce neuroinflammatory response and ischemic brain damage.^97^

A growing number of preclinical studies have confirmed Tregs as a promising post‐stroke immunotherapy strategy. However, Tregs‐related treatments may have some disadvantages. First, the intense anti‐inflammatory effect of Tregs might further inhibit the already suppressed immune system, resulting in undesirable side effects. For example, cancer‐bearing stroke mice exhibit accumulated Tregs within the tumor and exacerbated neuroinflammation, but the depletion of Tregs does not further aggravate ischemic brain injury and neuroinflammation in these mice.^97^ Second, the negative effects of Tregs in secondary thrombosis cannot be ignored. Finally, the potential toxicity or side effects of Tregs stimulators for the in vivo expansion of Tregs need to be further evaluated.

## 7 CONCLUSION

It is undeniable that Tregs can highly modulate the immune system after stroke, but the mechanisms are complex and many questions remain to be explored. The secondary thrombosis caused by non‐immune functions of Tregs and the influence of individual patient differences (e.g., gender, age, complications, and severity of disease) on Treg function all deserve further study to better measure the advantages and disadvantages of Tregs in ischemic stroke and to provide reference for clinical treatment.

## CONFLICT OF INTEREST

None.

## Data Availability

Data sharing not applicable to this article as no datasets were generated or analyzed during the current study.
